# Case Report: *Chlamydia psittaci* pneumonia complicated by Guillain-Barré syndrome detected using metagenomic next-generation sequencing

**DOI:** 10.3389/fcimb.2022.1070760

**Published:** 2023-01-23

**Authors:** Changquan Fang, Limin Xu, Jiarong Tan, Hongyi Tan, Junhong Lin, Ziwen Zhao

**Affiliations:** ^1^ Department of Pulmonary and Critical Care Medicine, Huizhou Central People’s Hospital, Huizhou, Guangdong, China; ^2^ Department of Geriatrics, Huizhou First People’s Hospital, Huizhou, Guangdong, China; ^3^ Department of Pulmonary and Critical Care Medicine, Guangzhou First People’s Hospital Affiliated to South China University of Technology, Guangzhou, Guangdong, China

**Keywords:** *chlamydia psittaci* pneumonia, psittacosis, Guillain-Barre syndrome, metagenomic next-generation sequencing, antibiotic therapy

## Abstract

Psittacosis and Guillain-Barré syndrome are both rare clinical diseases with low incidence, and their combination has rarely been reported. Here, we report a case of *Chlamydia psittaci* pneumonia combined with Guillain-Barré syndrome. The patient initially presented with high fever, difficulty breathing, and fatigue. Chest computerised tomography indicated large consolidation opacities in both lungs. Metagenomic next-generation sequencing clearly identified the pathogen as *C. psittaci*. The patient’s fever subsided after targeted antibiotic treatment, but difficulty breathing and fatigue worsened, and the patient developed symmetric limb numbness and weakness. Lumbar puncture, electrophysiological examination, and clinical characteristics were suggestive of Guillain-Barré syndrome, and the symptoms improved after treatment with human immunoglobulin. The results of this study suggest that metagenomic next-generation sequencing is useful for the rapid diagnosis of pulmonary infectious agents. Psittacosis is closely associated with the development of Guillain-Barré syndrome; however, more cases are needed to support this conclusion, and early targeted antibiotic treatment, immunotherapy, and basic supportive treatment are essential for improving outcomes.

## 1 Introduction

Psittacosis is a rare infectious zoonotic disease caused by *Chlamydia psittaci*, the source of which is primarily birds and poultry. Humans contract psittacosis primarily through inhalation of aerosols containing *C. psittaci* (Tantengco, 2022). This disease is a systemic infection with predominantly pulmonary involvement, typically presenting with high fever, chills, fatigue, headache, muscle aches, and pulmonary infiltrative lesions, as well as central nervous system complications, including meningitis, cranial nerve palsy, transverse myelitis, and Guillain-Barré syndrome (GBS) in a small number of patients (Beeckman and Vanrompay, 2009; Zhang et al., 2020). GBS is an autoimmune peripheral neurological disorder manifesting as symmetrical acute flaccid paralysis of the limbs, and pathogens such as *Campylobacter jejuni*, Zika virus, *Chlamydia*, and encephalitis B virus have been reported to be associated with this disease (Van den Berg et al., 2014; Wang et al., 2020). The combination of *C. psittaci* pneumonia and GBS has rarely been reported due to the difficulty in diagnosing psittacosis and its low incidence (Hogerwerf et al., 2017). Here, we report for the first time a case of *C. psittaci* pneumonia with concomitant GBS diagnosed using metagenomic next-generation sequencing (mNGS), aiming to increase awareness of this disease among clinicians.

## 2 Case presentation

**Figure 1:**
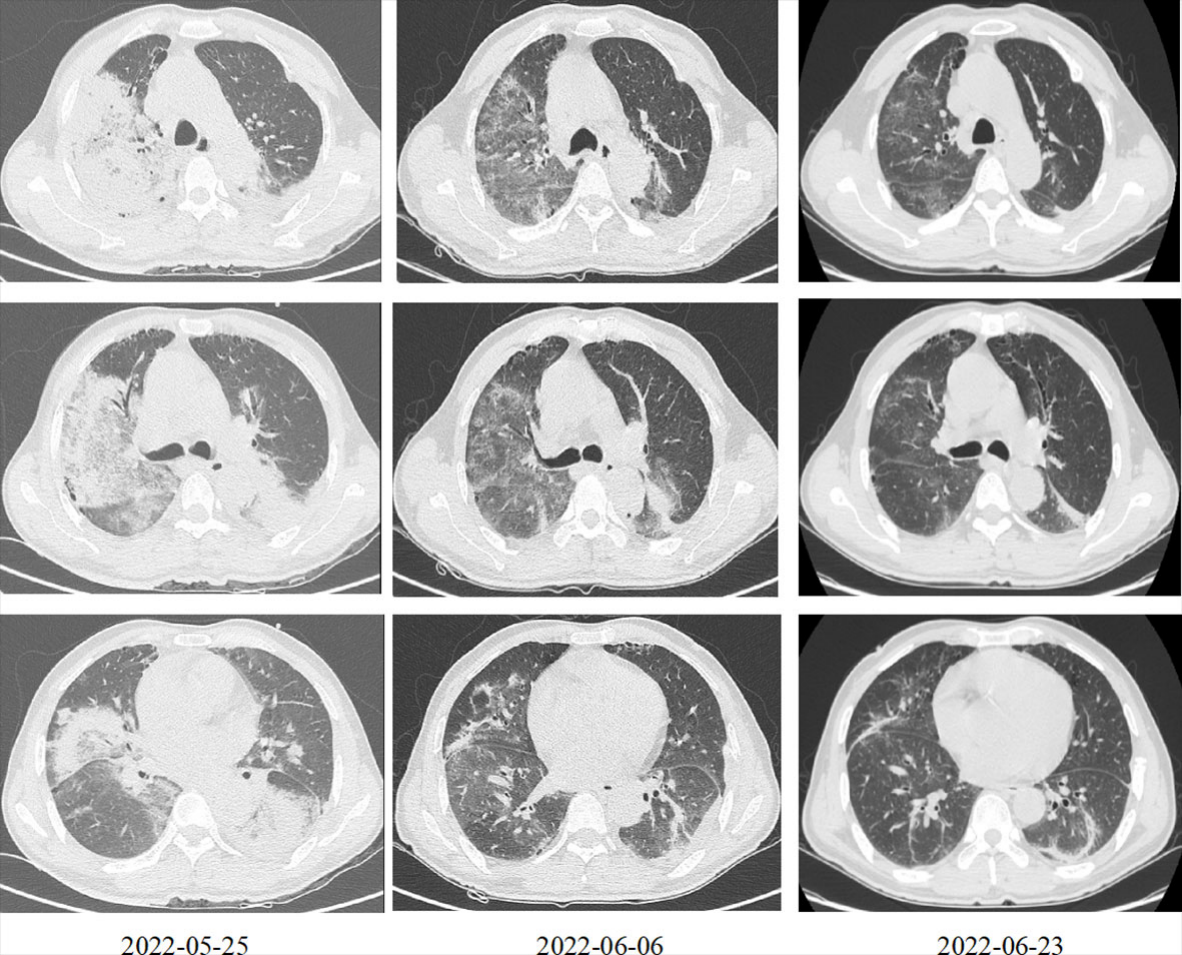
Chest computed tomography at different time points after treatment. March 25, 2022: Bilateral multiple exudative foci in the lungs, large consolidation opacities, and a small amount of bilateral pleural effusion. June 6, 2022: The exudative foci of both lungs were less absorbed than before. June 23, 2022: The exudative foci in both lungs were further absorbed as compared to before.

The patient was a 58-year-old male, who was a farmer by profession and gave no history of previous illness. He was admitted to the hospital on May 26, 2022 with a chief complaint of fever and cough for 1 week and difficulty breathing for 2 days. One week before admission, the patient developed a fever, with a maximum temperature of 40.0°C, with no obvious cause, accompanied by chills, poor appetite, fatigue, headache, and muscle aches, as well as intermittent cough and a small amount of sputum, which did not improve significantly after oral cefuroxime and acetaminophen treatment. Two days prior to admission, the patient had difficulty breathing, poor appetite, and fatigue that were more severe than before, so he sought treatment at our emergency department. Complete blood count revealed white blood cell (WBC) counts of 11.2 × 10^9^/L, a neutrophil percentage of 92.5%, C-reactive protein levels of 195.4 mg/L, and procalcitonin levels of 5.3 ng/mL. Chest computerised tomography (CT) indicated bilateral multiple exudative foci in the lungs, large consolidation opacities, and a small amount of bilateral pleural effusion (**Figure 1**). Diagnosis of pulmonary infection was considered, and combined meropenem and levofloxacin treatment was administered. However, this was ineffective and the patient’s body temperature fluctuated around 39.0°C; therefore, the patient was admitted for further diagnosis and treatment.

Physical examination revealed a body temperature of 39.0°C, pulse of 95 beats/min, respiratory rate at 35 breaths/min, blood pressure of 123/75 mmHg, and fingertip pulse oximetry at 92% (5 L/min oxygen by face mask). The patient was lucid and could answer questions. A palpable enlargement of any superficial lymph nodes throughout the body was not observed. The patient was short of breath, and coarse breath sounds in bilateral lungs and wet rales in bilateral lower lungs were heard. The heart rate was 95 beats/min and rhythmic, and no murmur was heard in each valve auscultation area. Muscle strength and tone in both upper limbs were normal. Muscle strength in both lower limbs was grade 4, and muscle tone was slightly reduced. The ankle jerk reflex was slightly weakened. No pathological signs were elicited.

Furthermore, laboratory tests indicated that the patient was negative for SARS-CoV-2 nucleic acid, as tested by real-time polymerase chain reaction (RT-PCR) and serology (IgM-ELISA) of respiratory pathogens (*Legionella pneumophila*, *Chlamydia pneumoniae*, *Mycoplasma pneumoniae*, *Coxiella burnetii*, adenovirus, respiratory syncytial virus, influenza A virus, influenza B virus, and parainfluenza virus), negative on the Widal and Weil-Felix tests, and negative on blood and sputum cultures. Other laboratory test results are shown in **Table 1**.

**Table 1:** Results of laboratory tests of the patient at different times.

Laboratory test	Normal range	First day of hospitalisation	After 3 days of targeted anti infection therapy	One day before discharge
Blood routine				
WBC (×10^9^/L)	4–10	9.6	6.7	8.1
Neutrophil (%)	40–75	92.3	63.2	65.7
LYM (×10^9^/L)	1.1–3.2	0.15	0.88	2.1
Inflammatory index				
C-reactive protein (mg/L)	0–5	250	99.5	5.35
Procalcitonin (ng/mL)	0–0.05	4.7	0.87	0.07
Interleukin-6 (pg/mL)	0–7	350.2	170.8	3.2
Biochemical indexes				
ALT (U/L)	9–50	105	64	39
AST (U/L)	15–40	239	68	35
CK (U/L)	50–310	615	221	90
LDH (U/L)	109–245	759	328	106
D-dimer (mg/L)	0–500	9900	3840	460
BUN (mmol/L)	2.9–8.2	8.0	6.6	5.0
Scr (μmol/L)	62–106	81.0	39.7	49.0
Lactic acid (mmol/L)	0.5–2.2	3.15	2.69	1.0
BNP (pg/mL)	0–100	220.0	144.9	52.5
High-sensitivity troponin T (ng/mL)	14–100	40.1	20.6	12.3

ALT, alanine aminotransferase; AST, aspartate aminotransferase; BNP, brain natriuretic peptide; BUN, blood urea nitrogen; CK, creatine kinase; LDH, lactate dehydrogenase; LYM, lymphocyte count; Scr, serum creatinine; WBC, white blood cell.

After admission, the patient was given combined meropenem and moxifloxacin treatment, oseltamivir treatment, hepatoprotective agents, and high-flow nasal cannula oxygen therapy. The patient was critically ill, and empirical antibacterial treatment was ineffective. To identify the causative pathogen, 10 mL of bronchoalveolar lavage fluid was collected *via* bronchoscopy on day 2 after admission and sent to Darui Diagnostics (Guangzhou, China) for mNGS. In the laboratory, DNA was extracted using the Ion AmpliSeq kit (Thermo Fisher Scientific), DNA libraries were constructed using the Da An Gene Universal Library Construction kit (Da An Gene Co., Ltd.), and sequencing was performed using the DA8600 proton high-throughput sequencing system (Ascendas Gene). On May 29, mNGS results indicated the presence of *C. psittaci* (sequence number 1891) and herpes simplex virus type I (sequence number 2). When asked about his medical history, the patient replied that he had been exposed to live chickens several times during the 2 weeks before disease onset; thus diagnosis of severe *C. psittaci* pneumonia was considered. Doxycycline combined with moxifloxacin was then used for anti-infection treatment. After 72 h of treatment, his body temperature gradually returned to normal, and laboratory tests indicated improvements in inflammation and organ function indicators (**Table 1**). However, difficulty breathing and fatigue did not improve significantly, so respiratory support was changed to non-invasive assisted ventilation. During this period, the complement system, electrolytes, anti-nuclear antibodies, anti-double-stranded DNA antibodies, anti-neutrophil cytoplasmic antibodies, myositis antibodies, thyroid function, and tumour biomarker tests did not reveal any significant abnormalities. On June 6, the patient developed numbness and weakness of the extremities, difficulty in holding objects and unstable standing, slurred speech, choking on drinking water, and difficulty swallowing. Neurological examination revealed conscious, unclear articulation, grade 3 proximal muscle strength, grade 4 distal muscle strength in both upper limbs, grade 3 muscle strength in both lower limbs, slightly reduced muscle tone, decreased deep sensation, and loss of tendon reflexes in the limbs, while no pathological signs were elicited. The patient exhibited symmetric limb weakness and loss of tendon reflexes, and thus, GBS was not excluded.

Head CT and Magnetic resonance imaging did not indicate any significant abnormalities. Chest CT indicated that the exudative foci of both lungs were less absorbed than before (**Figure 1**). Venous Doppler ultrasound of the upper and lower limbs did not indicate any thrombosis. Cerebrospinal fluid examination on June 7 revealed protein levels of 1176 mg/L, a WBC of 3 × 10^6^/L, and negative cerebrospinal fluid culture. Electromyography showed neurogenic peripheral neuropathy in the upper and lower limbs (predominantly demyelination damage), as well as abnormal F waves in the limbs (**Tables 2**, **3**). Taken together, the patient’s clinical symptoms, signs, and examination results were diagnostic of *C. psittaci* pneumonia combined with GBS. In addition to treatment for *C. psittaci* pneumonia, the patient was given intravenous human immunoglobulin at 0.4 g·kg^-1^·d^-1^ for 5 days, supplemented with nutritional nerve and rehabilitation therapy. After 5 days of targeted treatment, the patient’s limb numbness, fatigue, and difficulty breathing improved significantly, and the non-invasive assisted ventilation was gradually discontinued in favour of oxygen *via* nasal cannula. On June 23, chest CT indicated that the exudative foci in both lungs were further absorbed (**Figure 1**), and the inflammation and functional indicators of each organ were generally normal (**Table 1**). The patient was discharged the following day. At the time of discharge, the patient was stable, could stand and walk with assistance, had clear speech, did not have choking or swallowing difficulties, had grade 4 muscle strength in all four limbs, and tendon reflexes could be elicited. At follow-up 1 month after discharge, the patient was generally well, did not have respiratory distress, and could walk independently.

**Table 2:** Motor nerve conduction study.

Nerve	Latency (ms)		Amplitude (micro V)		Conduction Velocity (m/s)		F Wave	
	Right	Left	Right	Left	Right	Left	Right	Left
Median	15.8	11.8	0.4	0.3	45	35	Absent	Absent
Ulnar	2.8	3.4	3.4	2.3	52	54	–	–
Peroneal	4.2	11.0	1.1	0.4	47	64	–	–
Tibial	6.7	7.7	0.8	0.9	50	63	Absent	Absent

**Table 3:** Sensory nerve conduction study.

Nerve	Latency (ms)		Amplitude (micro V)		Conduction Velocity (m/s)	
	Right	Left	Right	Left	Right	Left
Median	Absent	Absent	Absent	Absent	Absent	Absent
Ulnar	2.1	3.1	2.1	4.7	48	34
Peroneal	Absent	Absent	Absent	Absent	Absent	Absent
Tibial	Absent	Absent	Absent	Absent	Absent	Absent

## 3 Discussion

*C. psittaci* is a Gram-negative, obligate intracellular parasitic pathogen, with severe pathogenicity (Knittler and Sachse, 2015). *C. psittaci* enters the human body *via* the respiratory tract and rapidly divides and proliferates, spreads through the blood, invades the reticuloendothelial system, and produces lesions in the lungs, liver, kidney, haematological system, and neuromuscular system (Knittler and Sachse, 2015; Zhang et al., 2020). Therefore, its clinical manifestations vary greatly and usually appear 5–14 days after infection. The primary symptoms include nonspecific respiratory symptoms, such as high fever, chills, fatigue, dry cough, and difficulty breathing, as well as symptoms of extrapulmonary organ involvement, including headache, muscle aches, and mental disturbances. Most patients have normal WBC counts and elevated creatine kinase and transaminases, and the primary manifestation of chest CT is consolidation (Li et al., 2022; Yang et al., 2022a; Yang et al., 2022b). In this case, the lungs and extrapulmonary organs, including the liver, neuromuscular system, and haematological system, were involved, and the patient had obvious fatigue and loss of tendon reflexes, which were inconsistent with the symptoms of infection, poisoning, or myositis, thus raising the possibility of secondary central nervous system pathology.

mNGS is a high-throughput sequencing technology and can be used to directly sequence all nucleic acids in clinical specimens without any prior assumptions of the causative pathogen, thus providing significant reference value for the diagnosis of new, complex, mixed, or unknown pathogens (Li et al., 2021). The number of detected sequences in the mNGS report refers to the number of sequences matched with the pathogen (Li et al., 2021). In the present case, two pathogens, herpes simplex virus type I and *C. psittaci*, were detected using mNGS. The possibility of colonisation was considered due to the extremely low number of herpes simplex virus type I sequences and the lack of compatibility with clinical manifestations. *C. psittaci* is a rare intracellular parasitic bacterium, and if its DNA sequence is detected using mNGS, the possibility of psittacosis should be considered (Li et al., 2021). Since the lung lesions were significantly absorbed after targeted anti-psittacosis treatment, the diagnosis of *C. psittaci* pneumonia was confirmed. Tetracyclines are the preferred treatment for psittacosis, followed by macrolides and quinolones (Balsamo et al., 2017). Because the intracellular concentration of quinolone antibiotics is relatively low, their clinical efficacy is lower than those of tetracyclines and macrolides (Butaye et al., 1997). In the present case, the patient was initially treated with moxifloxacin; however, the efficacy was poor. The patient’s condition improved after addition of doxycycline.

GBS is an acute inflammatory peripheral neuropathy closely associated with infection. It has a low incidence, at approximately 0.81–1.89/100,000 per year, and is more common in male patients. Acute inflammatory demyelinating polyneuropathy (AIDP) is the most common subtype of GBS and is typically characterised by (1) a history of frequent antecedent infection in many cases, (2) acute symmetric limb weakness and decreased or absent tendon reflexes in the limbs, (3) albuminocytological dissociation of the cerebrospinal fluid, and (4) electrophysiological examination suggestive of peripheral nerve demyelination (Van der Berg et al., 2014; Leonhard et al., 2019). In the present case, *C. psittaci* sequences were detected in the patient using mNGS, and the clinical manifestations were consistent with the characteristics of AIDP. Taken together with blood potassium, thyroid function, and electromyography results, periodic paralysis and myositis could be excluded; therefore, the diagnosis of *C. psittaci* pneumonia combined with GBS was confirmed. In the present case, high fever and difficulty breathing were the primary manifestations at disease onset, and AIDP-related symptoms were the primary manifestations in the middle and late stages of the disease. Grattan and Berman (1982) reported two patients who presented with weakness in the limbs as the primary symptoms; the initial presentation of *C. psittaci* pneumonia combined with GBS differed from the present case. However, all of these patients developed GBS secondary to respiratory tract infection 2–3 weeks after initial infection. *Chlamydia pneumoniae* belongs to the same genus of *Chlamydia* as *C. psittaci*, and studies have found that *M. pneumoniae* infection is closely associated with the development of GBS (Koike et al., 2021; Wong et al., 2021).

The pathogenesis of GBS secondary to *C. psittaci* pneumonia remains unclear. Previous studies have suggested a possible mechanism in which certain proteins in *C. psittaci* have sequences similar to those of peripheral nerve tissue proteins, and *C. psittaci* infection leads to autoantibody production, which can cause complement-mediated nerve injury in peripheral nerves due to antibody-antigen interaction, thereby damaging the peripheral, spinal, and brain nerves (Van den Berg et al., 2014; Koike et al., 2021; Shahrizaila et al., 2021). *C. psittaci* infection can lead to cellular immune dysfunction and systemic inflammation, massive release of proinflammatory cytokines such as IL-1, IL-6, and TNF-α, and over-activation of macrophages (Zhang et al., 2022).The pathogenesis of GBS is also due to the involvement of a variety of immune-cell subsets and a complex network of cytokines (Zhang et al., 2013).Therefore, secondary GBS caused by *C. psittaci* infection may be due to the fact that pathogen infection enhances the autoimmune response and causes the immune system to attack its own peripheral nerves. Because both psittacosis and GBS present with weakness or pain in the limbs, they are not easily distinguishable at early stages. However, if the weakness or pain worsens progressively, and especially if significant weakness and pain persist even after effective control of infection, it is necessary to be vigilant for the possibility of secondary GBS.

Treatment of GBS primarily involves etiological treatment, immunotherapy, and basic supportive treatment (Van den Berg et al., 2014). The onset of GBS is primarily associated with pathogenic infection, and early identification of the pathogen and targeted antibiotic treatment is effective in improving organ failure caused by the inflammatory response. The patient in the present case developed respiratory failure and hepatic insufficiency rapidly after the onset of the disease. After mNGS examination for pathogen identification, targeted treatment against psittacosis was administered, and the patient’s organ insufficiency improved. Khatib et al. (2017) reported a patient who eventually died from severe pneumonia secondary to *Streptococcus pneumoniae* infection due to delayed diagnosis and treatment. Intravenous immunoglobulin is a major type of immunotherapy for GBS that reduces neurological damage by binding autoantibodies, neutralising pathogenic autoantigens, and blocking the interaction between autoantibodies and autoantigens. The recommended dosage in adults is 0.4 g·kg^-1^·d^-1^ for 5 consecutive days (Van den Berg et al., 2014; Liu et al., 2018; Svačina et al., 2019). The response rate after immunoglobulin treatment was found to correlate with clinical outcomes, and the short-term outcomes of patients that started with intravenous immunoglobulin within 1 week were significantly better than those of patients that started treatment after 1 week (Jin et al., 2021). Grattan et al. (1982) reported a case of psittacosis complicated by GBS. Although targeted anti psittacosis, mechanical ventilation, and glucocorticoids were adopted, the patient died because of no intravenous immunoglobulin or plasma exchange. The above case suggests that a targeted treatment of GBS is the key to successful treatment. In addition, attention needs to be paid to GBS-related complications, such as respiratory paralysis and autonomic dysfunction, which must be addressed with supportive treatment as necessary (Van den Berg et al., 2014; Leonhard et al., 2019). After the patient had been diagnosed with GBS, he was promptly treated with human immunoglobulin, ventilator-assisted respiration, and neurotrophins. His condition improved 5 days after treatment, and he was discharged from the hospital with good recovery after timely treatment.

In conclusion, the results of this study suggest that mNGS is beneficial for early diagnosis of pulmonary infection pathogens, that GBS can be secondary to *C. psittaci* infection, and that early targeted antibiotic therapy, immunotherapy, and basic supportive therapy can improve patient outcomes.

## Data availability statement

The original contributions presented in the study are included in the article/supplementary material. Further inquiries can be directed to the corresponding authors.

## Ethics statement

The studies involving human participants were reviewed and approved by the Ethics Review Committee of Huizhou Central People’s Hospital. The patients/participants provided their written informed consent to participate in this study.

## Author contributions

CF and LX drafted the manuscript. JL and ZZ contributed substantially to its revision. HT collected the data. JT undertook review and editing. All authors contributed to the article and approved the submitted version.
